# Thyroid autoantibodies in adults with acquired binocular diplopia of unknown origin

**DOI:** 10.1038/s41598-020-62451-8

**Published:** 2020-03-25

**Authors:** Haeng-Jin Lee, Seong-Joon Kim

**Affiliations:** 1 Department of Ophthalmology, Seoul National University College of Medicine, Seoul, South Korea; 2 Seoul Artificial Eye Center, Seoul National University Hospital Clinical Research Institute, Seoul, South Korea

**Keywords:** Ocular motility disorders, Thyroid diseases

## Abstract

Patients with acquired adult-onset strabismus mainly present with binocular diplopia. Although cranial nerve palsies are reportedly the most common cause of binocular diplopia in adults, thyroid disease can also cause diplopia. In patients with thyroid-associated ophthalmopathy, upper lid retraction and proptosis are the most common initial findings, but diplopia could be the first manifestation. So far, there has been little information on the diagnostic value of thyroid autoantibodies in patients with strabismus. Therefore, we examined adults with acquired binocular diplopia from 2008 to 2016 and evaluated the presence of thyroid autoantibodies and the relationship between thyroid autoantibody status and clinical characteristics in adults with acquired binocular diplopia. Thyroid autoantibody tests were performed for all patients, unless other causes of diplopia were identified. Fifty one (39%) of 132 patients were positive for thyroid autoantibodies. In the thyroid autoantibody-positive (TAb+) group, microsomal autoantibodies, thyroid-stimulating hormone receptor antibodies, thyroglobulin antibodies, and thyroid-stimulating antibodies were observed in 30, 27, 12, and 7 patients, respectively. The vertical deviation and grade of duction limitation were greater in the TAb+ group. The presence of ocular torsion was 15.5% and 39.5% in the TAb− and TAb+ groups, respectively. Thyroid autoantibody evaluation may be helpful in adults with idiopathic acquired binocular diplopia.

## Introduction

Patients with acquired adult-onset strabismus mainly present with binocular diplopia. Although cranial nerve palsies are reportedly the most common cause of binocular diplopia in adults, thyroid-associated ophthalmopathy (TAO) can cause binocular diplopia^1–3^. Therefore, physicians should suspect the presence of TAO, if a patient presents with diplopia and a history of thyroid disease such as Graves’ disease, hypothyroidism, or thyroid cancer.

In patients with TAO, upper lid retraction and proptosis are the most common initial findings, but diplopia could be the first manifestation in 16.7% of patients^4^. Previous studies have reported that the treatment of hyperthyroidism does not appear to influence the course of TAO^5,6^. On the other hand, thyroid-stimulating hormone (TSH) receptor Ab levels have been reported to be correlated with disease activity. Therefore, assessment of thyroid autoantibodies, especially TSH receptor antibodies, has been suggested, which can guide the management of thyroid eye disease, helping to prevent the serious manifestations^7–12^. However, there is little information on the diagnostic value of thyroid autoantibody status in patients with strabismus. The main purpose of this study was to evaluate the presence of thyroid autoantibodies in adults with acquired binocular diplopia of unknown etiology, and the clinical characteristics in patients with thyroid autoantibodies. In addition, we investigated that thyroid autoantibody measurement may be helpful for the diagnosis of patients with binocular diplopia.

## Results

### Patient demographics and clinical characteristics

Of the 667 patients screened for eligibility, 271 patients with a history of ocular surgery, 113 patients whose age of onset of binocular diplopia was uncertain or those who were diagnosed before the age of 17 years, 110 patients with 3 brain lesions, 21 with underlying diseases, and 20 patients with a history of ocular trauma were excluded. Thus, 132 patients were included in the analysis. Of 132 patients, 16 patients had been previously diagnosed with thyroid disease. Except for two patients with exophthalmos and upper lid retraction, rest of patients only had binocular diplopia without other clinical sign of TAO.

The mean age was 53.8 ± 16.1 years and the mean duration of binocular diplopia was 32.8 ± 48.4 months. The mean angle of horizontal deviation was 12.4 ± 12.4 PD and the mean angle of vertical deviation was 5.5 ± 8.0 PD. One hundred and nine patients (82.6%) were euthyroid. Among the patients who were positive for thyroid autoantibodies, 30 patients had microsomal autoantibodies, 27 had TSH receptor antibodies, 12 had thyroglobulin antibodies, and 7 had thyroid-stimulating antibodies (Table 1).

**Table 1:** Demographics and clinical characteristics of total 132 patients with binocular diplopia.

Variable	Values	Range
Age, years, mean ± SD	53.8 ± 16.1	17.0–81.0
Sex (male:female)	57:75	
Duration of diplopia, months, mean ± SD	32.8 ± 48.4	0.0–340.0
**Refractive error, diopters, mean ± SD**		
Right eye	−1.43 ± 2.89	−9.75–5.50
Left eye	−1.35 ± 2.78	−9.75–5.50
**Thyroid function state, numbers (%)**		
Hyperthyroidism	4 (3.0%)	
Subclinical hyperthyroidism	7 (5.3%)	
Euthyroidism	109 (82.6%)	
Hypothyroidism	12 (9.1%)	
**Thyroid autoantibodies, numbers (%)**		
Microsomal Ab	30 (22.7%)	
TSH receptor Ab	27 (20.5%)	
Thyroglobulin Ab	12 (9.1%)	
Thyroid stimulating Ab	7 (5.3%)	
Horizontal strabismus, numbers (%)	123 (93.2%)	
Vertical strabismus, numbers (%)	74 (56.1%)	
Horizontal angle of deviation, PD, mean ± SD	12.4 ± 12.4	0.0–74.0
Vertical angle of deviation, PD, mean ± SD	5.5 ± 8.0	0.0–35.0
Duction limitation, numbers (%)	52 (39.4%)	
Oblique muscle dysfunction, numbers (%)	12 (9.1%)	
Ocular torsion, numbers (%)	31 (22.8%)	

SD, standard deviation; Ab, antibody; PD, prism diopters.

### Comparison of the clinical characteristics of patients with binocular diplopia based on the thyroid autoantibody status

Fifty-one patients (38.6%) were positive for thyroid autoantibodies. In the TAb+ group, mean values of microsomal autoantibodies, TSH receptor antibodies, thyroglobulin autoantibodies, and thyroid-stimulating antibodies were as follows: 1226.4 ± 1631.1 U/ml, 5.6 ± 7.8 IU/L, 1172.9 ± 2102.0 IU/L, and 367.7 ± 168.4%. Thyroid dysfunction was more common in the TAb+ group than that in the TAb− group. The angle of horizontal deviation was 11.3 ± 10.2 PD in the TAb− group and 14.1 ± 15.3 PD in the TAb+ group (*P* = 0.258). The angle of vertical deviation was 3.5 ± 5.7 PD and 8.7 ± 10.0 PD, in the TAb− and TAb+ groups, respectively (*P* = 0.001). Duction limitation was present in 22 patients (27%) in the TAb− group and 30 patients (59%) in the TAb+ group (*P* < 0.001). Ocular torsion was present in 11 patients (14%) in the TAb− group and in 20 patients (39%) in the TAb+ group (*P* = 0.001, Table 2).

**Table 2:** Comparison of clinical characteristics of patients with binocular diplopia according to the presence of thyroid autoantibodies.

	TAb− group (n = 81)	TAb+ group (n = 51)	*P* value
Age, years, mean ± SD	51.6 ± 18.2	57.4 ± 11.3	0.056^a^
Sex (male:female)	40:41	17:34	0.070^b^
Duration of diplopia, months, mean ± SD	31.1 ± 42.1	35.7 ± 57.6	0.604^a^
Thyroid function state, numbers (%)			<0.001^b^
Hyperthyroidism	1 (1.2%)	3 (5.9%)	
Subclinical hyperthyroidism	1 (1.2%)	6 (11.8%)	
Euthyroidism	77 (95.1%)	32 (62.7%)	
Hypothyroidism	2 (2.5%)	10 (19.6%)	
Horizontal strabismus, numbers (%)	75 (92.6%)	48 (94.1%)	0.516^b^
Vertical strabismus, numbers (%)	40 (49.4%)	34 (66.7%)	0.038^b^
Horizontal angle of deviation, PD, mean ± SD	11.3 ± 10.2	14.1 ± 15.3	0.258^a^
Vertical angle of deviation, PD, mean ± SD	3.5 ± 5.7	8.7 ± 10.0	0.001^a^
Duction limitation, numbers (%)	22 (27.2%)	30 (58.8%)	<0.001^b^
Grade of duction limitation, mean ± SD	−0.6 ± 0.2	−0.8 ± 0.4	<0.001^a^
Oblique muscle dysfunction, numbers (%)	7 (8.6%)	5 (9.8%)	0.526^b^
Ocular torsion, numbers (%)	11 (13.6%)	20 (39.2%)	0.001^b^

TAb− group, thyroid autoantibody-negative group; TAb+ group, thyroid autoantibody-positive group; SD, standard deviation; PD, prism diopters

^a^independent t-test, ^b^Fisher’s exact test.

### Comparison of clinical characteristics of thyroid autoantibody-positive patients according to the thyroid function

In the TAb+ group, thyroid function was normal in 32 patients and abnormal in 19 patients. There was no difference in age, sex, duration of diplopia, angle of deviation, or in duction limitation or ocular torsion between the two groups (Table 3). No differences were observed in the clinical characteristics according to positivity or negativity of each of the four autoantibody types.

**Table 3:** Comparison of clinical characteristics of patients who were positive for thyroid autoantibodies according to the thyroid function.

	Normal thyroid function (n = 32)	Abnormal thyroid function (n = 19)	*P* value
Age, years, mean ± SD	56.3 ± 10.5	59.3 ± 12.5	0.362^a^
Sex (male:female)	13:19	4:15	0.129^b^
Duration of diplopia, months, mean ± SD	44.3 ± 67.8	20.8 ± 30.0	0.172^a^
Horizontal strabismus, numbers (%)	31 (96.9%)	17 (89.5%)	0.309^b^
Vertical strabismus, numbers (%)	19 (59.4%)	15 (78.9%)	0.129^b^
Horizontal angle of deviation, PD, mean ± SD	12.8 ± 12.7	16.2 ± 19.2	0.454^a^
Vertical angle of deviation, PD, mean ± SD	7.9 ± 10.5	10.1 ± 9.20	0.451^a^
Duction limitation, numbers (%)	15 (46.9%)	15 (78.9%)	0.011^b^
Grade of duction limitation, mean ± SD	−0.7 ± 0.4	−1.0 ± 0.4	0.051^a^
Oblique muscle dysfunction, numbers (%)	3 (9.4%)	2 (10.5%)	0.623^b^
Ocular torsion, numbers (%)	11 (34.4%)	9 (47.4%)	0.224^b^

SD, standard deviation; PD, prism diopters

^a^independent t-test, ^b^Fisher’s exact test.

### Changes in eye movements during follow-up in patients with binocular diplopia according to thyroid autoantibody status

**Figure 1:**
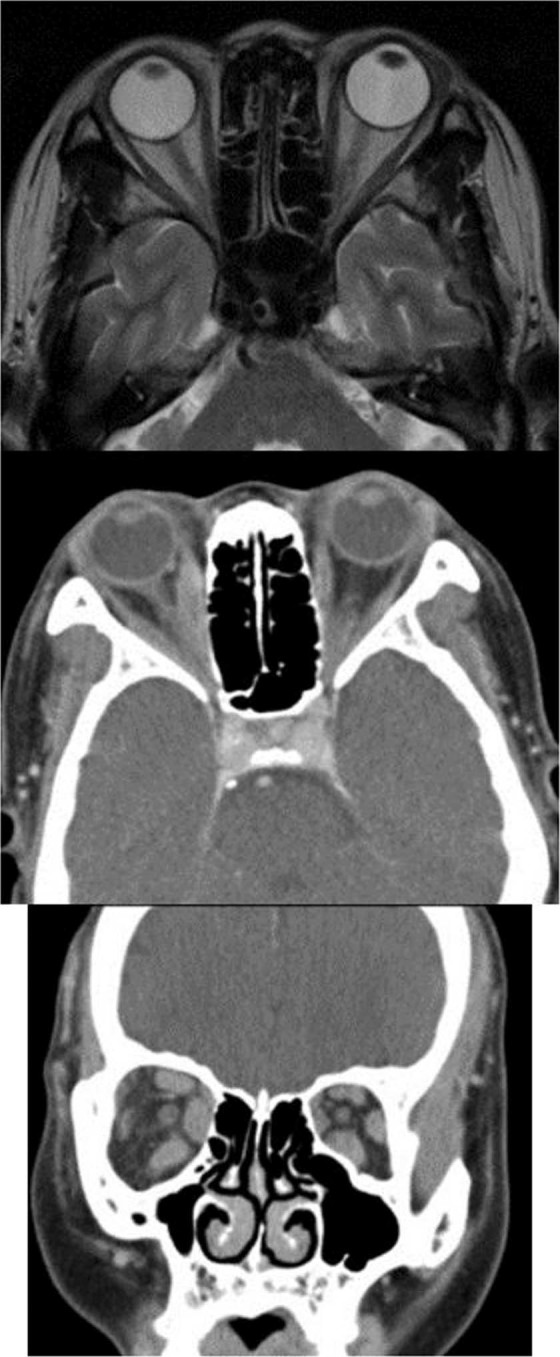
Change of extraocular muscle (EOM) in patient with thyroid autoantibodies during follow-up. The imaging scans of one patient who was positive for thyroid autoantibodies. (**a**) At first visit, he presented 6 prism diopters of horizontal deviation angle. There was no EOM limitation and thyroid function was normal. MRI presented no abnormal lesion in brain and normal thickness of extraocular muscle. (**b,c**) After 42 months of follow-up, diplopia was getting worse and patient was newly diagnosed with Graves’ disease. The EOM was significantly thickened. Change of horizontal deviation angle was 11 PD and 12PD of vertical deviation was newly presented.

Of a total of 132 patients, 109 were followed up for more than 3 months (67 in the TAb− group and 42 in the TAb+ group). During a mean follow-up of 15.6 ± 18.1 months, two patients in the TAb+ group were newly diagnosed with Graves’ disease and one was diagnosed with Hashimoto’s thyroiditis. The imaging scans of one patient are presented in Fig. 1. The change in the horizontal deviation angle was 3.3 ± 3.5 PD in the TAb− group and 4.4 ± 5.1 PD in the TAb+ group, while the change in the vertical deviation angle was 1.2 ± 1.8 PD in the TAb− group and 5.9 ± 8.1 PD in the TAb+ group (*P* = 0.227 and *P* = 0.001, respectively). The change in grade of duction limitation was 0.1 ± 0.1 in the TAb− group and 0.2 ± 0.3 in the TAb+ group (*P* < 0.001, Table 4).

**Table 4:** Comparison of eye movement change during follow up in patients with binocular diplopia according to the presence of thyroid autoantibodies.

	TAb− group (n = 67)	TAb+ group (n = 42)	*P* value
Follow-up period, months, mean ± SD	18.1 ± 17.5	20.2 ± 19.8	0.553^a^
Horizontal angle of deviation at final follow up, PD, mean ± SD	13.3 ± 12.4	16.0 ± 17.8	0.370^a^
Vertical angle of deviation at final follow up, PD, mean ± SD	4.0 ± 6.5	10.6 ± 12.8	0.004^a^
Grade of duction limitation at final follow up, mean ± SD	−0.6 ± 0.2	−0.3 ± 0.3	<0.001^a^
Change of horizontal angle of deviation, PD, mean ± SD	3.3 ± 3.5	4.4 ± 5.1	0.227^a^
Change of vertical angle of deviation, PD, mean ± SD	1.2 ± 1.8	5.9 ± 8.1	0.001^a^
Change of grade of duction limitation, mean ± SD	0.1 ± 0.1	0.2 ± 0.3	<0.001^a^

TAb− group, thyroid autoantibody-negative group; TAb+ group, thyroid autoantibody-positive group; SD, standard deviation; PD, prism diopters.

^a^Independent t-test.

## Discussion

The prediction and prevention of acquired binocular diplopia in adults is a challenging task often. Acquired binocular diplopia has several causes, among which cranial nerve palsies are the most common causes. TAO is also a cause of binocular diplopia in adults and is most commonly associated with Graves’ disease. However, it is also occasionally observed in patients with Hashimoto’s thyroiditis, primary hypothyroidism, and thyroid cancer, and in patients who have undergone radiotherapy for the neck region^13^. If a patient with acquired binocular diplopia has been previously diagnosed with any of these thyroid diseases, it would be helpful to identify TAO as the cause of diplopia. If there is no history of thyroid disease, thyroid function and thyroid autoantibody tests are usually performed for the diagnosis of TAO.

Autoantibodies against thyroid gland antigens and the orbit might cross-react with both tissues. There are conflicting reports in the literature regarding the value of thyroid autoantibody tests in patients with thyroid disease^9,14–18^. Thyroid-stimulating antibody testing has been reported to be helpful for making a diagnosis of TAO in euthyroid patients and for the assessment of its severity^9,15^. In addition, some researchers asserted that new treatment modalities such as specific monoclonal antibodies, TSH-R antagonists, and other immunomodulatory agents show a promising outcome for Graves’ ophthalmopathy patients^19^. However, another study showed that the thyroid-stimulating antibody was not a marker for Graves’ ophthalmopathy^14^. Recently, Lin *et al*.^20^ reported that there were no significant predictors of strabismus following a diagnosis of TAO. Despite these controversial reports, thyroid autoantibodies are still considered valuable in the diagnosis and management of Graves’ disease^9,12,15,21^. However, most of the previous studies included patients with Graves’ disease and patients who had already been diagnosed with TAO, and their main clinical manifestations were exophthalmos and lid retraction. Moreover, risk factors such as cigarette smoking^22,23^, and exposure to radioactive iodine have been linked to development of TAO, but none of the risk factors have been clearly associated with strabismus^24^.

TAO usually causes a restrictive, incomitant, and vertical strabismus. Therefore, in patients without other clinical sign of TAO, restrictive pattern could be the clue to suspect the strabismus caused by thyroid disease. In addition, the most common affected muscles in TAO are the inferior and medial recti. Since vertical rectus muscle involves the ocular torsion, measurement of ocular torsion is helpful to manage the patients with TAO^25,26^. The present study included patients with binocular diplopia. After excluding the patients with a clear etiology of diplopia, 38.6% of patients were positive for thyroid autoantibodies. These patients had a greater angle of vertical deviation and a higher duction limitation grade and were more likely to have ocular torsion. This finding suggests that patients who tested positive for thyroid autoantibodies could present the strabismus seen in TAO.

Abnormal thyroid hormone levels were also more common in the TAb+ group. However, 62.7% of these patients had normal thyroid function. Although there was no difference in the clinical characteristics among euthyroid patients and those with abnormal thyroid function in the TAb+ group, the vertical angle of deviation and duction limitation grade increased during the follow-up. This suggests that patients who tested positive for thyroid autoantibodies should be followed up regularly, even if their thyroid function tests appear normal, in order to detect a change in the deviation angle or duction limitation. For example, a change in the deviation angle and thickening of the extraocular muscles were observed in the patient shown in Fig. 1. Our findings suggest the possibility of changes in the clinical condition in patients who are positive for thyroid autoantibodies. Therefore, thyroid autoantibodies could be helpful markers for the differential diagnosis of binocular diplopia in adults.

This study had some limitations, resulting mainly from its retrospective design. For example, patients lacking medical records were excluded. We were also unable to obtain information on the smoking history. Although all the ophthalmologic examinations were performed by one experienced physician, duction was subjectively graded. Moreover, the possibility of a false-positive or false-negative result of thyroid antibody assays should be considered. In terms of types of Ab assay, we could not find the differences in the clinical characteristics among four types of thyroid autoantibodies. Prospective studies with larger populations, including patients with a clear etiology of binocular diplopia and long-term follow-up are needed in the future.

Nevertheless, to the best of our knowledge, this was the first study to screen a considerably large number of adults for acquired binocular diplopia and report the relationship of the thyroid autoantibody status with strabismus. The angle of vertical deviation and grade of duction limitation were greater in the TAb+ group. Ocular torsion and thyroid dysfunction were more common in the TAb+ group. We assumed that there would be changes in strabismus in the patients in the TAb+ group, which necessitates regular follow-up. In conclusion, thyroid autoantibody status could be helpful for the diagnosis of patients with binocular diplopia.

## Methods

### Subjects

We retrospectively reviewed the medical records of adults (aged above 17 years) who were diagnosed with acquired binocular diplopia at Seoul National University Hospital between January 2008 and July 2017. Patients who had undergone ocular surgery for cataract or refractive surgery, patients with a history of ocular trauma, and those with underlying disease, such as myasthenia gravis or chronic progressive external ophthalmoplegia were excluded. Patients with strabismus caused by a brain lesion, such as a brain tumour, brain haemorrhage, or microvascular lesion, patients who were diagnosed with strabismus before the age of 17 years, and patients in whom the age of onset of diplopia was not documented were also excluded. The patients enrolled underwent laboratory investigations and brain and orbit magnetic resonance imaging. The study was approved by the Institutional Review Board (IRB) of Seoul National University Hospital in South Korea and the study protocol followed the tenets of the Declaration of Helsinki. The IRB waived the need for informed consent as it was retrospective chart review study.

### Thyroid autoantibody tests

Patients underwent thyroid function and thyroid autoantibody tests, unless other causes of diplopia were identified. Thyroid function tests included measurement of T3, T4, free T4, and TSH levels and were divided into four categories; hyperthyroidism, subclinical hyperthyroidism, euthyroidism, and hypothyroidism. Thyroid autoantibody tests were performed in all patients using the same assays: microsomal autoantibodies using chemiluminescent microparticle immunoassay (normal reference value 0–60 U/ml), TSH receptor antibodies using electrochemiluminescence immunoassay (normal reference value 0–1 IU/L), thyroglobulin autoantibodies using electrochemiluminescence immunoassay (normal reference value 0–100 U/ml), and thyroid-stimulating antibodies using bioimmunoassay. In case of thyroid-stimulating antibodies, the specimen-to-reference ratio (SRR%) was calculated according to the following formula: (mean specimen TSAb/mean reference TSAb)*100. Patient serum was considered positive if SRR was ≥ 140%. We divided patients according to their thyroid autoantibody test results into the thyroid autoantibody-positive (TAb+) group and the thyroid autoantibody-negative (TAb−) group. Factors including age, sex, duration of diplopia, and angle of deviation were compared between two groups.

### Ophthalmologic examination

At the initial visit, all patients underwent manifest and cycloplegic refraction. The refractive error was converted to the spherical equivalent value [in dioptres (D)]. Myopia was represented as negative numbers and hyperopia as positive numbers. Amblyopia was defined as a difference of two lines or more in Snellen acuity and excluded in this study. The angle of deviation was measured by alternate prism cover test with accommodative targets for fixation both at near (0.33 m) and at distance (6 m) and indicated as prism diopters (PD). The horizontal and vertical deviation angles were analyzed separately after measurement. Limitation of duction was measured using a five-point scale (0 to −4), from “0” (indicating no limitation with full duction) through “−3” (indicating an eye, which could reach the midline) to a maximum of “−4” (representing no movement beyond the midline, with the eye fixed in the opposite position). Presence of inferior/superior oblique muscle dysfunction such as overaction and underaction was also evaluated. All ophthalmologic examinations were performed by an experienced ophthalmologist (SJ-K).

Objective ocular torsion was assessed using fundus photography. Patients were asked to look at an internal fixation target in order to align their eyes at the primary position. Based on the position of the fovea, we considered the eye to be in extorsion when the fovea was located below the horizontal line at the lower edge of the optic disc. We considered the eye to be in intorsion, when the fovea was located above the horizontal line passing through the center of the optic disc^27^. Physiological torsion was defined by the fovea laying in the area between the center and the lower edge of the optic disc. The present study analyzed the presence of pathological ocular torsion.

### Statistical analysis

As the data showed normal distribution by Kolmogorov-Smirnov test, parametric methods including the independence t-test, Fisher’s exact test were used. Continuous variables are reported as mean ± standard deviation. *P* < 0.05 was considered statistically significant for all the tests. Statistical analyses were performed using SPSS software (version 23 for Windows; SPSS, Chicago, IL, USA).

## Data Availability

Data supporting the findings of the current study are available from the corresponding author on reasonable request.
